# Genetic Diversity and Infection Prevalence of *Biomphalaria pfeifferi* (Krauss, 1848), the Intermediate Snail Host of *Schistosoma mansoni* in Gezira State, Sudan

**DOI:** 10.3390/ijms26199567

**Published:** 2025-09-30

**Authors:** Arwa Osman, Peter S. Andrus, Yuan Fang, Ibrahim Elhassan, Xiaonong Zhou, Bakri Y. M. Nour, Liming Zhao

**Affiliations:** 1 State Key Laboratory of Bioreactor Engineering, East China University of Science and Technology, Shanghai 200237, China; y10180460@mail.ecust.edu.cn; 2 Blue Nile National Institute for Communicable Diseases, University of Gezira, Wad Madani P.O. Box 20, Sudan; 3 National Institute of Parasitic Diseases, Chinese Center for Disease Control and Prevention (Chinese Center for Tropical Diseases Research), Shanghai 200025, China; 4 School of Global Health, Chinese Centre for Tropical Diseases Research, Shanghai Jiao Tong University School of Medicine, Shanghai 200025, China; 5 Department of Immunology and Parasitology, Copenhagen University, 1172 København, Denmark; 6 Department of Parasitology, Endemic Diseases Institute, University of Khartoum, Khartoum 11115, Sudan; 7 Faculty of Medical Laboratories, University of Gezira, Wad Madani P.O. Box 20, Sudan

**Keywords:** genetic diversity, *Biomphalaria pfeifferi*, *Schistosoma mansoni*, mitochondrial population genetics, molecular xenomonitoring, Gezira State, Sudan

## Abstract

*Biomphalaria pfeifferi* snails serve as the major intermediate host for intestinal schistosomiasis in Sudan. The genetic structure and infection status of 163 *B. pfeifferi* collected from six localities in Gezira State, Sudan (East Gezira, Greater Wadmedani, Hasahisa, North Umelgura, South Gezira, and Managil) were characterized. Cytochrome oxidase subunit I (*COI*) and *16S* ribosomal RNA (*16S rRNA*) mitochondrial genes were used for *B. pfeifferi* molecular identification and genetic diversity investigation. *Schistosoma mansoni* infection was detected using the traditional cercarial shedding and molecular methods (Sm^F/R^ primers). Five *COI* haplotypes and ten *16S* haplotypes were identified, with haplotype diversity of 0.50 for *COI* and 0.11 for *16S*. High evolutionary divergence was observed between groups (Fst = 0.94) for the *COI*, and low genetic divergence (Fst = 0.04) for the *16S*, indicating genetic divergence among Sudanese *B. pfeifferi*, with the *16S* showing lower divergence than the *COI*, consistent with a post-bottleneck population expansion. Cercarial shedding detected an overall infection prevalence of 3.6% (8/219), with only two snails from Hasahisa shedding *S. mansoni* cercariae. The Sm^F/R^ primers revealed a higher infection prevalence of 7.4% (12/163), with all *S. mansoni* positive samples found at the Hasahisa site. Findings highlight the value of molecular diagnostic tools for accurate surveillance and emphasize the need for site-specific control strategies.

## 1. Introduction

The freshwater snail genus *Biomphalaria* (Gastropoda: Planorbidae) is widely distributed across sub-Saharan Africa, South and Central America, and the Middle East [1,2]. The disease disproportionately affects vulnerable groups, including preschool and school-aged children, certain occupational workers, women engaged in domestic activities involving contaminated water, and communities living in high-risk areas [3]. In Sudan, schistosomiasis has become a significant public health concern, and the disease is endemic in all states, except the Red Sea State, resulting in serious economic and social implications. The expansion of water resource development and increased population mobility have contributed to the disease spread and prevalence [4,5]. Gezira State, a major agricultural area in Sudan, has experienced a significant rise in schistosomiasis prevalence. This increase is partly linked to changes in crop rotation within the Gezira Irrigation Scheme and the development of the Managil Extension, which have limited access to sanitary services and safe water [5,6]. The interaction between schistosomiasis transmission and water resource development underscores the importance of understanding the dynamics of snail intermediate hosts and disease transmission [7].

All African *Biomphalaria* species are capable of transmitting *Schistosoma mansoni*, the primary causative agent of intestinal schistosomiasis in humans [1]. Among these, *B. pfeifferi* is considered the most important vector due to its high infection prevalence in field populations [1,8,9]. In addition to *S. mansoni*, African *Biomphalaria* species are also susceptible to a range of other trematode parasites [10]. For example, *Biomphalaria* snails in Sudan have been found infected with Amphistome, Echinostome, Furcocercous, and Xiphidiocercariae cercariae types [11,12].

Historically, snail identification relied on shell morphology and anatomical characteristics (e.g., radula and genitalia) to determine taxonomic differences between species [1,13]. To date, a total of 34 *Biomphalaria* species have been identified [1,2], with the African species being historically classified into four morphological groups: Alexandrina, Choanomphala, Pfeifferi, and Sudanica [14]. However, molecular phylogenies have since redefined these classifications, defining the African species as *B. camerunensis*, *B. pfeifferi*, and the Nilotic species complex (*B. alexandrina*, *B. angulosa*, *B. choanomphala*, *B. smithi*, *B. stanleyi*, and *B. sudanica*) [8,15,16,17]. Molecular techniques have been employed to improve the precision of species identification and to enhance our understanding of the population structure and genetic diversity of medically important snail species [18,19]. The application of molecular methods has greatly improved the accuracy of snail identification, thereby advancing our understanding of schistosomiasis epidemiology [20].

Numerous studies have investigated the phylogenetic structure of *B. pfeifferi* populations across sub-Saharan Africa using established genetic markers, such as the Cytochrome oxidase subunit I (*COI*) gene, *16S* ribosomal RNA (*16S rRNA*), and the Internal Transcribed Spacer regions (*ITS1* and *ITS2*) [2,8,21,22,23,24,25,26,27,28]. Similarly, several studies have investigated the prevalence of *S. mansoni* infection in *B. pfeifferi* populations, traditionally using the cercarial shedding method, which involves screening snails over a 35-day period [29]. However, this method detects only patent infections [30] and fails to detect pre-patent infections. To address this limitation, a variety of molecular xenomonitoring assays targeting different genes have been developed [23,28,31,32,33,34,35], enabling the detection of both patent and pre-patent infections. These molecular detection techniques offer high sensitivity for detecting low quantities of *S. mansoni* DNA in *Biomphalaria* spp. [32,35,36], enable simultaneous identification of multiple parasite species [37], and allow accurate species confirmation through genotyping [26].

In 2023, the prevalence of intestinal schistosomiasis in Gezira State was 8.6%, highlighting the ongoing public health burden and the urgent need for integrated control strategies. Accurate species-level identification and a thorough understanding of the genetic structure of *Biomphalaria* snails in endemic regions like Gezira State are essential for designing effective interventions, as both factors influence their susceptibility to *Schistosoma* infection and the dynamics of disease transmission. This study aims to investigate the genetic diversity and population structure of *B. pfeifferi* across multiple Gezira State localities using mitochondrial *COI* and *16S* markers to infer phylogenetic relationships with other African populations. Infection prevalence was assessed through both cercarial shedding and PCR-based (Sm^F/R^) methods. By integrating genetic and epidemiological data, this study provides insights into the diversity and infection dynamics of *B. pfeifferi* in Gezira State, with the aim of informing and strengthening evidence-based schistosomiasis control strategies.

## 2. Results

### 2.1. Species Identification and Phylogenetic Analysis

**Figure 1:**
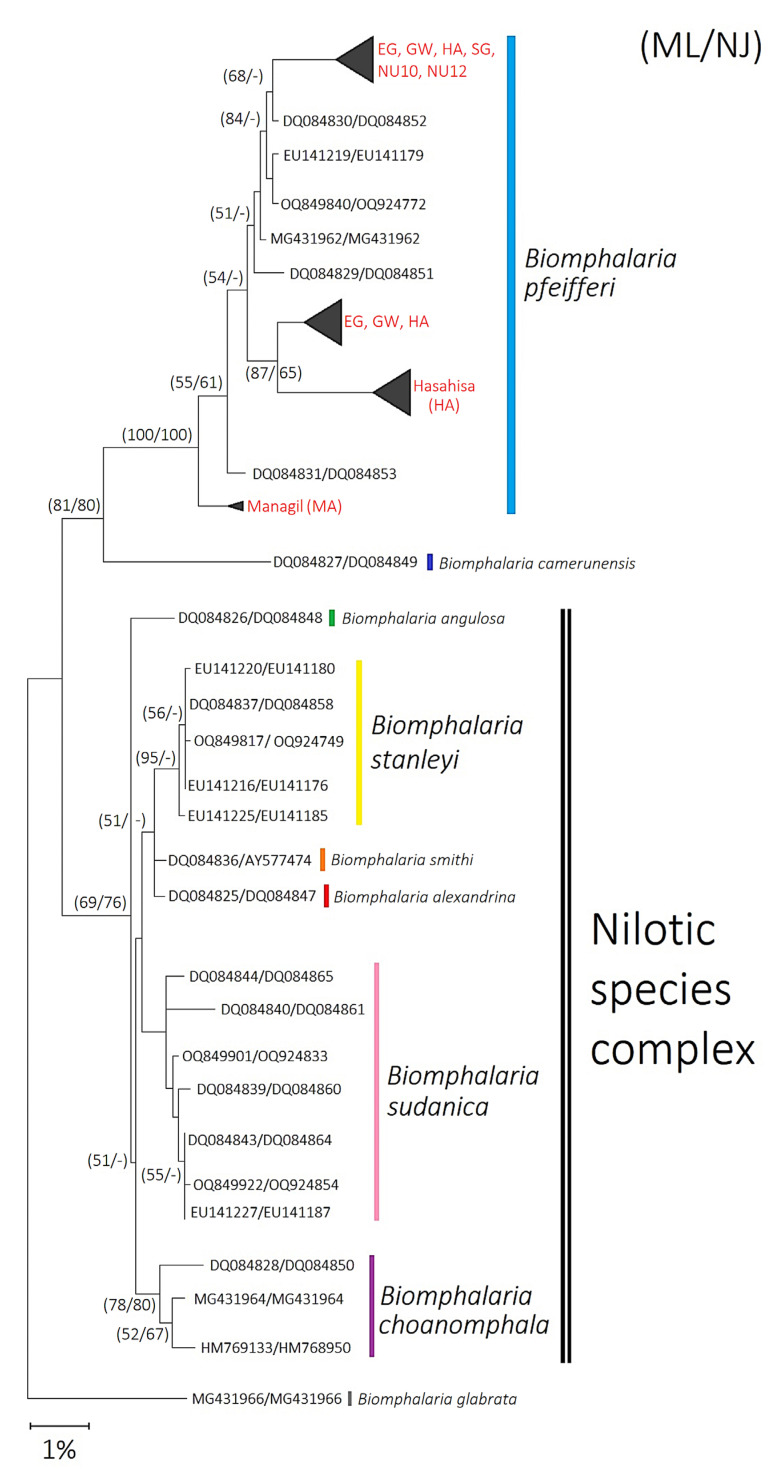
Maximum Likelihood and Neighbor-Joining consensus tree of African *Biomphalaria* species based on the concatenated *COI* (465 bp) and *16S rRNA* (322 bp) gene fragments. Phylogenetic reconstruction was conducted under the GTR+Γ model and rooted with *B. glabrata*. Red labels indicate *B. pfeifferi* sequences generated in this study. Numbers on branches represent bootstrap support values (1000 replicates) from Maximum Likelihood and Neighbor-Joining (ML/NJ) analyses; values < 50% are not shown. The scale bar corresponds to 1% sequence divergence.

Of the six locations surveyed, *Biomphalaria* snails were found at all locations but were present at only seven of the 30 sites (23.3%; Table 1). In total, 219 *Biomphalaria* snails were collected from these seven sites across Gezira State, Sudan. Of those 219, 163 had their DNA extracted and were confirmed as *B. pfeifferi* through the phylogenetic analysis of the *COI* and *16S* gene fragments (Figure 1). The remaining 56 snails were also identified as *B. pfeifferi* based on key morphological characteristics. The Maximum Likelihood (ML) and Neighbor-Joining consensus phylogenetic tree analysis of the concatenated *COI* and *16S* dataset confirmed that all samples from Gezira clustered with reference *B. pfeifferi* sequences, supported by a 100% bootstrap value (Figure 1; Supplementary Figure S1). Among the Gezira populations, the Managil (MA) samples exhibited the highest genetic divergence, while moderate divergence was also observed in samples from Hasahisa (HA), East Gezira (EG), Greater Wadmedani (GW), South Gezira (SG), and both North Umelgura sites (NU1 and NU3; Figure 1).

### 2.2. Haplotype Analysis, Diversity Parameters, and Neutrality Tests

After alignment, the amplified fragments of the *COI* and *16S* gene fragments were 527 bp and 387 bp in length, respectively. For the *COI* gene, five haplotypes were identified. The predominant haplotype, H1, was found across four localities, East Gezira, South Gezira, and North Umelgura (NU1 and NU3), and accounted for 59.5% of all samples (97/163). Haplotype H2 occurred at three localities: Hasahisa, East Gezira, and Greater Wadmedani, comprising 37.4% (61/163) of the samples. The remaining haplotypes were more localized, with H3 being unique to East Gezira, H4 to Hasahisa, and H5 to Managil. East Gezira exhibited the highest haplotype richness with three haplotypes, followed by Hasahisa with two, while the remaining sites only had single haplotype (Table 1). In contrast, the *16S* gene revealed greater haplotype diversity, with ten haplotypes found across all sampled populations. Haplotype H1 was the most widespread, present in all seven localities, and constituted 93.8% of the total sequences (153/163). The remaining minor haplotypes were exclusive to Hasahisa (H2–H6), North Umelgura-1 (H7–H9), and North Umelgura-3 (H10). Hasahisa showed the highest haplotype diversity (with six haplotypes), followed by North Umelgura-1 (four haplotypes) and North Umelgura-3 (two haplotypes). The overall haplotype diversity (Hd) was 0.50 (±0.022) for *COI* and 0.11 (±0.035) for *16S*. The average number of nucleotide differences (k) was 5.38 for *COI* and 2.05 for *16S*. Lastly, nucleotide diversity (π) was estimated at 0.01 (±0.001) for *COI* and 0.008 (±0.003) for *16S* (Table 1).

Neutrality analyses revealed distinct patterns between the *COI* and *16S* gene datasets (Table 1). For the *COI* gene, Tajima’s D showed a negative but non-significant value (−0.867), suggesting weak evidence of population expansion or purifying selection at the population level. Similarly, Fu’s Fs was positive (13.62) and non-significant, which may indicate a recent population bottleneck or balancing selection, although the signal was weak. In contrast, the *16S* gene exhibited a statistically significant negative Tajima’s D value (−2.71, *p* < 0.01), indicating stronger evidence for recent population expansion or purifying selection. At the population level, *COI* data from East Gezira showed a significantly negative Tajima’s D value (−2.36, *p* < 0.02), supporting expansion in this population. However, Fu’s Fs remained positive and non-significant in both the overall and East Gezira datasets. For the *16S* gene, Hasahisa displayed the highest nucleotide diversity (0.035 ± 0.01) and haplotype diversity (Hd = 0.31 ± 0.11) among all sites, with a significantly negative Tajima’s D (−1.84, *p* < 0.05), indicating a recent expansion. It also showed a high, positive Fu’s F value (8.825). Though not statistically significant, this may suggest the presence of multiple divergent lineages at the site. Conversely, North Umelgura-1 (NU1) also showed relatively high haplotype diversity (0.25 ± 0.1), with a significantly negative Tajima’s D (−2.425, *p* < 0.01) and strongly negative Fu and Li’s D and F values, indicating a strong signal of recent expansion or purifying selection. Interestingly, North Umelgura-3 (NU3) showed limited diversity with only two haplotypes, and its Fu’s F value was negative (−1.21), but not statistically significant. Several other sites (Greater Wadmedani, South Gezira, and Managil) were monomorphic across both genes, consistent with low genetic variability, possibly due to recent colonization or population bottlenecks (Table 1).

### 2.3. Haplotypes Network Inference

**Figure 2:**
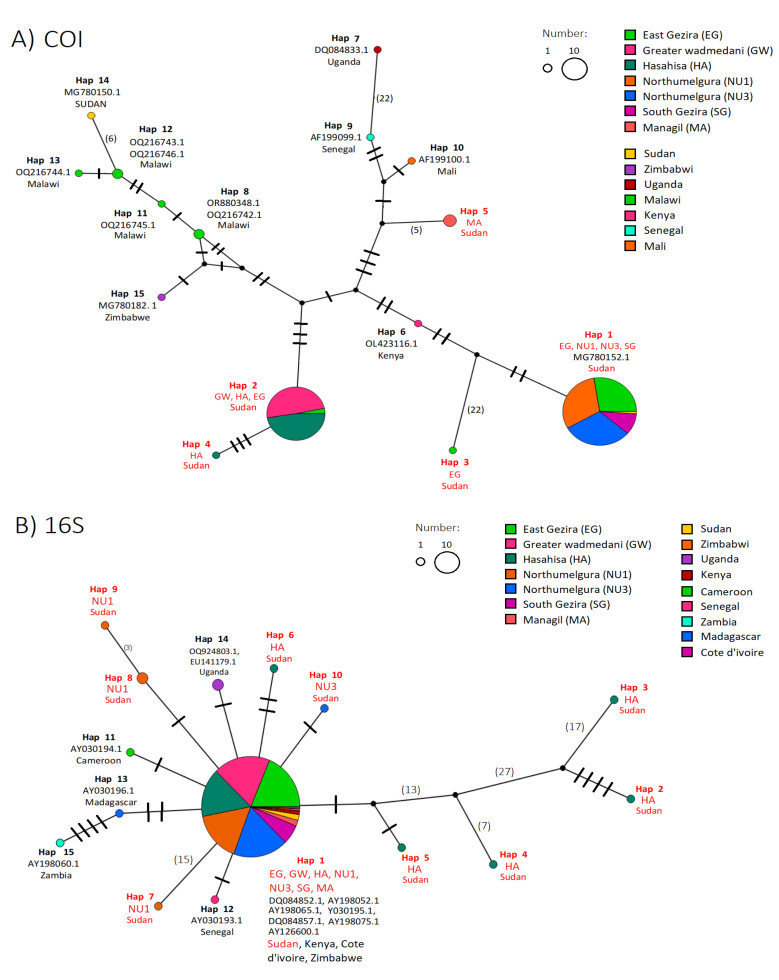

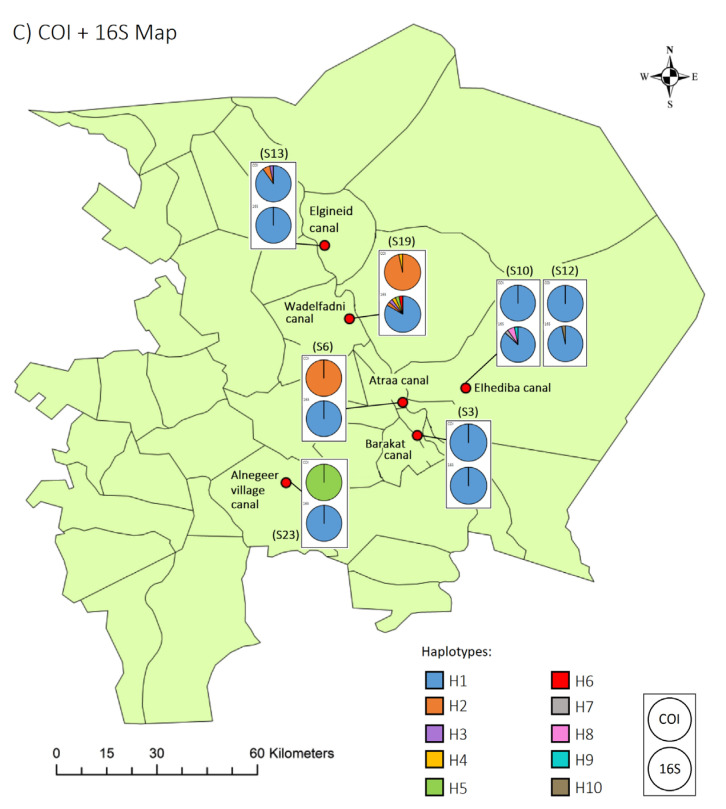
TCS haplotype networks and haplotype distribution of Sudanese *Biomphalaria pfeifferi* based on partial *COI* (527 bp) and *16S* (387 bp) gene fragments. (**A**) *COI* haplotype network and (**B**) *16S* haplotype network generated using PopART. Circle sizes are proportional to haplotype frequency. Hatch marks and numbers on branches indicate mutational steps between haplotypes; branch lengths are not to scale. Each color represents a different geographic origin (Green = EG, Pink = GW, Dark green = HA, Orange = NU1, Blue = NU3, Purple = SG, Red = MA), and haplotypes identified in this study are written in red. (**C**) Haplotype distribution map showing the geographic composition of haplotypes for both markers (*COI*: top; *16S*: bottom) across the seven Gezira State sites. Colors correspond to distinct haplotypes. Map created in ArcGIS v10.5 (Esri, CA, USA; https://www.arcgis.com/).

For the *COI* gene fragment, haplotype H1 was found across four localities in the Gezira state, and was distinct from all other African haplotypes, clustering only with a previously sequenced Sudanese *B. pfeifferi* sequence (MG78152; Figure 2A). Likewise, the remaining haplotypes (H2–H5) did not cluster with any other African haplotypes, suggesting local differentiation. For the *16S* gene, haplotype H1, was distributed across all seven Gezira sites, and clustered with sequences from multiple African countries, including Kenya (DQ084852.1, AY198052.1), Côte d’Ivoire (AY198065), Sudan (Y030195, DQ084857, AY198075), and Zimbabwe (AY126600; Figure 2B). This widespread clustering indicates low divergence and the dominance of this haplotype across Africa. However, similar to the *COI*, the remaining *16S* haplotypes (H2–H10) did not cluster with any other African haplotypes. These results highlight the higher resolution of the *COI* gene in detecting local genetic differentiation, while the *16S* gene reveals broader haplotype conservation across African *B. pfeifferi* populations. Analysis of haplotype distribution across the seven sampling sites (Figure 2C; Supplementary Table S1) revealed pronounced spatial structuring. For the *COI*, Elgineid (S13) exhibited the greatest haplotype diversity, with Wadelfadni (S19), Atraa (S6), and Alnegeer village (S23) being made up of haplotypes H2, H4, and H5 respectively. For the *16S* dataset, diversity was highest at Wadelfadni (S19) and Elhediba (S10, S12), where additional haplotypes (H2–H10) were detected at low frequencies (Figure 2C). Overall, these results indicate that haplotype H1 is highly conserved and dominant throughout Gezira, while localized differentiation is evident at specific sites, with *COI* offering finer resolution of site-specific variation than *16S*.

### 2.4. Genetic Divergence and Population Differentiation

Estimates of evolutionary divergence among *COI* haplotypes, calculated using the Maximum Composite Likelihood model, revealed substantial genetic variation. The highest divergence values were found between H1 (the most abundant haplotype) and H3 (0.053), H2 and H3 (0.068), and between H3 and H4 (0.074) and H5 (0.075) (Supplementary Table S2). The *16S* dataset exhibited even greater divergence, between H1 and H3 (0.192) and between H3 and H7 (0.220; Supplementary Table S2). The *COI* gene fragment revealed 41 polymorphic sites (Table 1), and pairwise population Fst values ranged from 0 to 1. The overall Fst was extremely high (0.94), indicating very strong genetic differentiation, and the Nm value was very low (0.03), suggesting minimal gene flow (Table 2A). Nearly all pairwise comparisons involving Managil (MA), Greater Wadmedani (GW), and Hasahisa (HA) exhibited very high differentiation (Fst > 0.85). Conversely, East Gezira (EG), North Umelgura sites (NU1 and NU3), and South Gezira (SG) shared low Fst values (0.02) among each other, indicating weak differentiation and relatively recent shared ancestry. The *16S* gene fragment showed higher overall polymorphism (84 sites) but low overall Fst (0.04) and high gene flow (Nm = 5.72; Table 2B). Most pairwise comparisons fell within the low differentiation range (Fst < 0.05), particularly between East Gezira and all other sites. Notably, comparisons involving Hasahisa (HA) yielded moderate Fst values (0.04–0.05), suggesting slightly increased genetic structuring in that locality, which also exhibited the highest haplotype diversity for *16S*.

### 2.5. Analyses of Molecular Variation (AMOVA)

The AMOVA results revealed strong genetic differentiation among the seven populations based on the *COI* gene fragment, with limited gene flow. The majority of genetic variation (84.6%) was attributed to differences among populations, while only 15.4% of the variation occurred within populations (Table 3). Although the fixation index (ΦST) was not statistically significant (*p* > 0.001), Wright’s criteria suggest that FST > 0.25 represents high divergence, while FST > 0.05 represents low divergence. Therefore, the *COI* gene has a very high fixation index (ΦST = 0.90), indicating significant evolutionary divergence and clear population structuring. FST values greater than 0.25 denote high genetic differentiation, which aligns well with the observed ΦST of 0.90 for *COI*. Although the term “FST” is often used interchangeably with “ΦST” in haplotype data, the distinction is important in this context. Here, ΦST clearly demonstrates strong population subdivision and restricted gene flow (Nm = 0.03). In contrast, the *16S* gene fragment exhibited very low genetic differentiation among populations, with only 3.6% of the variation attributed to differences among populations and 96.4% occurring within populations (Table 3). The fixation index was correspondingly low (ΦST = 0.04), consistent with weak population structure and relatively high gene flow (Nm = 5.72). Together, these results suggest that while *COI* haplotypes are strongly structured among populations, *16S* haplotypes show much less differentiation, reflecting differing evolutionary dynamics or rates of gene flow for the two markers.

### 2.6. Cercariae Shedding and PCR Infection

**Figure 3:**
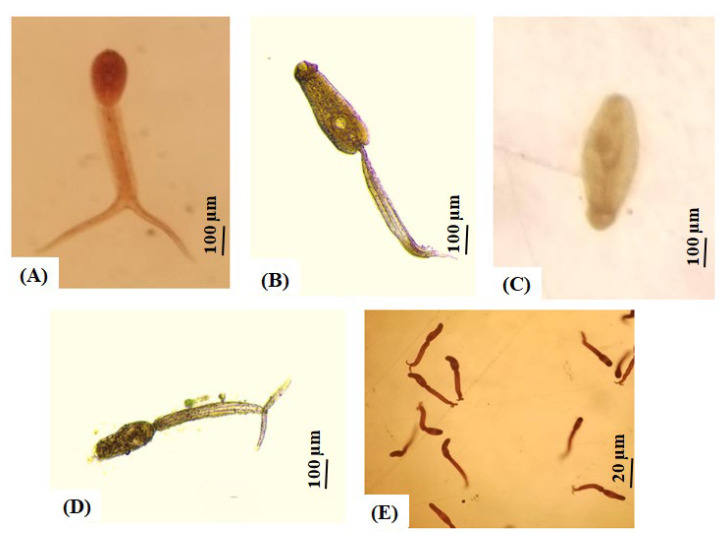
Types of cercariae shed by *Biomphalaria pfeifferi* across different localities in Gezira State: (**A**) *Apharyngostrigea* sp.; (**B**) *Echinostome* type; (**C**) *Cotylomicrocercous* type; (**D**,**E**) *Schistosoma mansoni* cercariae. All photos were taken under 10× magnification.

The overall prevalence of *B. pfeifferi* shedding cercariae across the seven surveyed sites was 3.6% (8/219), with North Umelgura showing the highest infection rate at 16.6% (Table 4). Among the eight shedding snails, four were co-infected with *Echinostome* spp. and *Cotylomicrocercous* spp. Cercariae (NU3), one was infected with *Echinostome* spp. (NU3), one was infected with *Apharyngostrigea* spp. (EG), and two were infected with *S. mansoni* (HA; Table 4; Figure 3). No snails were found shedding cercariae at the remaining sites (SG, GW, NU1, and MA), snails shedding *S. mansoni* cercariae were only found at Hasahisa (2/39; Table 4; Figure 3D,E).

To further assess *S. mansoni* infection, DNA extracts from *B. pfeifferi* snails were screened using Sm^F/R^ primers. The Sm^F/R^ primers detected *S. mansoni* DNA in 12 of 163 snail extracts tested (7.4%). All 12 of the Sm^F/R^ PCR-positive snails were from the Hasahisa site, and this included the two shedding snails and ten non-shedding (pre-patent) snails (Supplementary Figure S2). When sequenced, the Sm^F/R^ gene fragment matched (97% query coverage and 99.35% sequence identity) with multiple *S. mansoni* reference sequences in GenBank, including AY157173 [38], HE601625 [39], MK085970 [40], and OK310887, confirming the presence of *S. mansoni* DNA in Hasahisa.

## 3. Discussion

Mitochondrial genes, particularly *COI* and *16S* rRNA, are well-established markers for detecting genetic patterns linked to demographic variation within a species [41]. The high value of haplotype diversity (Hd) compared to nucleotide diversity (π) suggests closely related haplotypes and recent population expansion after a bottleneck [42]. We used neutrality tests to assess the demographic expansion of *B. pfeifferi* populations. The results collectively support the expansion hypothesis, as Tajima’s D (*COI*) and the significantly negative Fu and Li’s D and F values (both *COI* and *16S*) provide evidence of a past bottleneck. Additionally, Fuʼs Fs test also supports demographic expansion. However, some populations (e.g., Hasahisa, East Gezira, and North Umelgura-1) showed positive values, suggesting possible historical selection of alleles present in many individuals within these populations [43].

Next, Fst values were calculated to assess allele frequency variation among the studied populations, reflecting genetic differences and the degree of population substructure [44]. The results revealed strong divergence in *COI*, with highly significant Fst values (>0.5) and low gene flow among most populations, particularly in Managil (H5), which exhibited a unique SNP profile. However, the small sample size (n = 3) prevents a definitive conclusion about the isolation of Managil. The high divergence in *COI* among populations, combined with low gene flow, is consistent with previous studies on *B. pfeifferi*, *B. glabrata*, and *B. choanomphala* [2,8,43,45]. Conversely, the high homogeneity in the *16S* gene (low Fst = 0.041) indicates minimal differentiation among populations but high variation within populations, consistent with a previous study in Uganda [46]. The levels of divergence for each genetic marker varied, although the collection sites were geographically distant (each from a different locality), except for North Umelgura (NU1 and NU3). Moreover, the presence of distinct individuals in the Hasahisa, East Gezira, and North Umelgura localities aligns with several studies reporting that *Biomphalaria* spp. and *Bulinus* spp. populations are typically more genetically similar at nearby sites, although distinct haplotypes may still occur at individual locations [45,47,48].

The genetic structure of *Biomphalaria* spp. populations is influenced by many factors, such as self-fertilization, environmental conditions, habitat fluctuations, host type, and parasitic infection [1,19,25,28,36,46,49]. Bottlenecks in these snails are often linked to drastic environmental changes, such as shifts in climate, drought, flooding, food scarcity, and the use of molluscicides [2,25,49,50,51]. All of our snail samples were collected from non-perennial canals within the Gezira Irrigation System, which are subject to seasonal fluctuations such as droughts and occasional flooding. These environmental disturbances likely lead to periodic local population extinctions followed by recolonization, creating repeated genetic bottlenecks. Such events can reduce genetic diversity within populations while simultaneously increasing genetic divergence among populations, as surviving or recolonizing snails may carry only a subset of the original alleles. In addition, *B. pfeifferi* populations typically show a preference for self-fertilization, resulting in limited genetic variation [52,53]. Moreover, limited dispersal further increases genetic divergence by restricting gene flow [2,54]. In addition, the observed low genetic diversity in mitochondrial genes may reflect the adaptation of oxidative systems to environmental stressors [55,56]. The low level of genetic variation in *16S* is attributed to inbreeding or changes in habitat, leading to population subdivision [1,46,57]. It has been previously reported that *Biomphalaria* populations are subdivided based on variation in the *16S* gene [28,49,58,59,60,61].

Assessing infection prevalence in snail populations through cercarial shedding remains a cornerstone of traditional field-based schistosomiasis epidemiology. However, its key limitation is that it only detects patent infections (snails actively shedding cercariae), while failing to identify pre-patent cases [62,63]. Consequently, molecular diagnostics provide a more accurate representation of snail-mediated transmission, particularly in areas where both patent and pre-patent infections occur [64,65]. In our study, only 2 of 219 (0.9%) *B. pfeifferi* snails were found to be infected with *S. mansoni* using the cercarial shedding method, both from Hasahisa. In contrast, PCR detection identified infection in 12 of 163 *B. pfeifferi* DNA extracts (7.4%), which were all from Hasahisa. A meta-analysis by Hailegebriel et al. (2020) estimated the pooled prevalence of *S. mansoni* infection in *Biomphalaria* snails across Africa at 5.6%, indicating that the infection prevalence in our samples is lower than generally expected in natural snail populations [66]. Also, our results are consistent with previous studies demonstrating that molecular methods are more sensitive than cercarial shedding in detecting both pre-patent and patent infections [32,67,68]. These results confirm that relying solely on cercarial shedding for schistosomiasis screening can underestimate the true prevalence of *S. mansoni* infection [69]. This highlights the greater sensitivity of molecular methods and the failure of the cercarial shedding method to accurately identify infection in field snails, which can give the false impression of low or even no transmission in an area [62].

Despite their simplicity and effectiveness, molecular methods are limited by high costs and logistical challenges, such as requiring specialized equipment and stable conditions. Moreover, molecular methods may overestimate transmission risk, as not all PCR-positive snails go onto shed cercariae. For example, Lu et al. (2016) [67] found only 60% of PCR-positive *B. pfeifferi* snails proceeded to shed cercariae, likely due to the failed encapsulated sporocysts still being detected. Molecular detection methods are also highly susceptible to contamination from DNA templates or reagents, exhibit limited efficiency in detecting extremely low DNA concentrations, and require stringent reaction conditions, particularly precise annealing temperature control, for optimal performance [70,71]. In resource-limited developing countries, DNA extraction and molecular xenomonitoring of snails poses significant challenges. Despite these constraints, molecular methods remain essential for accurate schistosomiasis diagnosis and for studying the genetic diversity of snail host populations in Sudan and across Africa.

In terms of genetic diversity, *S. mansoni* was only detected in one site, Hasahisa. This could impact the diversity of snails haplotypes in this area, as discussed previously [25,60]. However, we noticed that infected individuals were not limited to a specific haplotype, despite the genetically distinct individuals in this population (six haplotypes in the *16S* gene and two in the *COI*), suggesting no clear genetic barrier to infection. Similar findings were noted in snails infected with different trematode cercariae at the two locations in the North Umelgura locality. This aligns with previous studies in *Biomphalaria* spp., where parasitism may shape population structure without affecting their susceptibility to schistosomes infection [45,49,60,72]. Notably, Hasahisa is one of the most genetically diverse sites, alongside East Gezira (EG) and the North Umelgura sites (NU1 and NU3). This pattern is consistent with Andrus et al. (2023, 2025), who found that *Biomphalaria* populations with higher amounts of *COI* and *16S* haplotypes harbor more *S. mansoni* infections than those with fewer haplotypes [28,36]. This may result from greater migration of infected snails introducing new parasites, alongside a wider range of host genotypes that increase compatibility with *S. mansoni*. In contrast, populations with lower genetic diversity may have reduced parasite compatibility and less introduction of infection due to limited migration. Notably, non-*S. mansoni* infection was also found in East Gezira and North Umelgura, further supporting the role of genetic diversity in shaping trematode transmission dynamics among *Biomphalaria* populations.

## 4. Materials and Methods

### 4.1. Study Area, Site Selection, and Sample Collection

**Figure 4:**
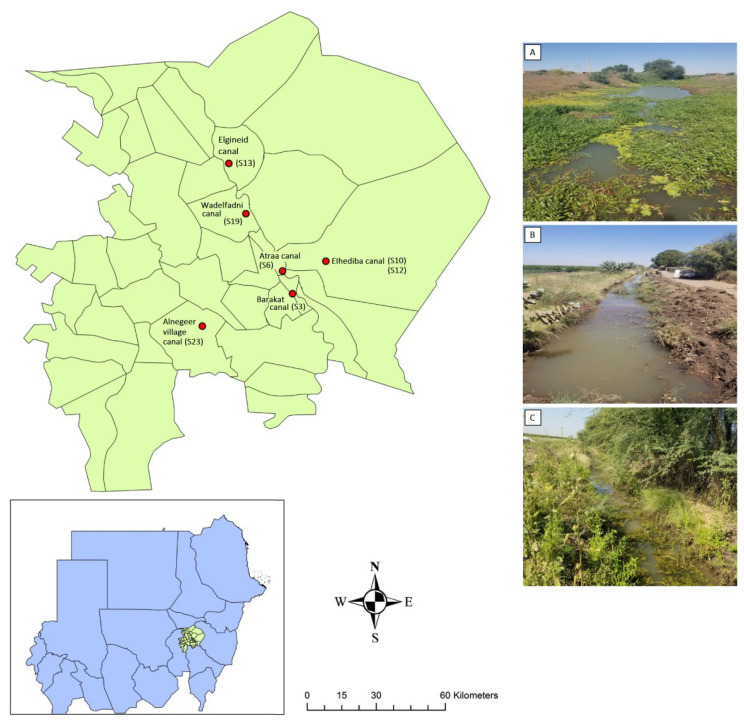
Map of the six collection locations in Gezira State, Sudan, highlighting examples of the non-perennial canal sites: (**A**) Greater Wadmedani, Atraa canal; (**B**) Hasahisa, Wadelfadni canal; and (**C**) South Gezira, Barakat canal.

Gezira State, central Sudan, lies between the Blue and White Nile rivers (13–14.2° N, 32.5–33° E) and supports the continent’s largest gravity-fed irrigation scheme (~7 million acres) via canals supplied from Sennar Dam (Figure 4) [73]. The scheme’s network of open earth-lined canals and associated ditches provides ideal habitats for *Biomphalaria* snails and frequent human contact points that sustain schistosomiasis transmission [74,75,76]. Sites were surveyed between December 2022 and March 2023, with *B. pfeifferi* snails being collected from six locations: East Gezira (EG), South Gezira (SG), Hasahisa (HA), Greater Wadmedani (GW), Managil (MA), and North Umelgura (NU; Figure 4, Table 5). These sites were chosen based on water-body type (e.g., minor canal, stream, field canal), observed human and animal activity, and historical surveys of urinary and intestinal schistosomiasis [77,78]. At each location, five sampling sites (30 sites in total) were surveyed. However, *B. pfeifferi* snails were found at only seven of the 30 sites (Table 5). All seven sites were non-perennial canals, experiencing intermittent water levels that fluctuate according to the farming season. GPS coordinates were recorded on site and mapped in ArcGIS 10.5 (Esri, Redlands, CA, USA; Figure 4). Two trained collectors inspected each site for 15–20 min, scooping along the canal edge and vegetation with a metal mesh net. *Biomphalaria* snails were transported in canal water to the Medical Entomology and Vector Control Department Laboratory in the Blue Nile National Institute for Communicable Diseases, University of Gezira, Sudan for sorting and were then identified morphologically [79,80].

### 4.2. Snail Screening (Cercaria Shedding)

Field collected snails (n = 219) were individually placed in 3 mL of tap water within glass bottles (40 × 20 mm) and exposed to artificial light for 3 h (9:00 am–12:00 pm) to induce cercarial shedding. Cercariae were examined under a dissection microscope (Olympus, Tokyo, Japan) and identified as either non-mammalian or mammalian (either human-related or non-human-related) cercariae using morphological descriptions by Frandsen & Christensen [10] and Schell [81]. Snails were labeled as either shedding-positive or shedding-negative and then preserved in 95% ethanol and stored at −20 °C until processing for molecular analysis at the National Institute of Parasitic Diseases, China CDC, Shanghai, China.

### 4.3. DNA Extraction

Where possible, 30 snails were randomly selected from each site for DNA extraction. If fewer than 30 snails were available at a site, all snails were included (see Table 5). The snail shells were crushed individually, and the head–foot tissue were isolated and preserved in 95% ethanol and stored at −20 °C prior to molecular identification and infection detection. Prior to DNA extraction, tissue was soaked overnight in ddH_2_O to remove residual ethanol and then air-dried for 4 hrs. Genomic DNA was extracted using the Qiagene DNeasy Blood and Tissue Kit (LOT: 175021295, Cat No. 69506, QIAGEN GmbH, Hilden, Germany) following the manufacturer’s instructions. The extracted DNA samples were stored at −20 °C until further analysis.

### 4.4. Molecular Identification

Two mitochondrial gene fragments were used to identify the *Biomphalaria* DNA extracts, the cytochrome oxidase subunit I (*COI*), and the *16S* ribosomal RNA (*16S* rRNA). The *COI* was amplified using the LCO1490 and HCO2198 primers developed by Folmer et al. [82], and the *16S* was amplified using the 16Sar and 16Sbr primers developed by Palumbi et al. [83] (Table 6). All polymerase chain reactions (PCRs) were performed in a final volume of 25 μL, comprising 13 μL of TianGEN 2× Taq PCR Mix (0.1 U Taq polymerase/μL, 500 μM dNTPs, 20 mM Tris-HCl, 100 mM KCl, 3 mM MgCl_2_), 7.5 μL of nuclease-free water, 1 μL each of forward and reverse primers (10 μM), and 2.5 μL of genomic DNA template. PCR cycling conditions for the *COI* primers consisted of an initial denaturation at 94 °C for 2 min, followed by 35 cycles of 94 °C for 30 s, 45 °C for 30 s, and 72 °C for 1 min, with a final extension at 72 °C for 10 min. For the *16S* primers, conditions were similar except the annealing temperature was 48 °C and each denaturation step was 94 °C for 1 min. Primer details and annealing temperatures followed those described by [8,24,84]. PCR products were visualized on a 1.5% agarose gel stained with GelStain C056 (1000× code #GS1014, Lot #P40910) and visualized under UV light. The PCR products were purified and sequenced using Beijing QingKe Biotechnology Company Ltd. (Shanghai, China).

#### 4.4.1. Sequence Alignment and Phylogenetic Analysis

Nucleotide sequences were visually edited using Bioedit software v7.7.1 [85]. The sequences from each site were grouped together and aligned using Clustal W in Molecular Evolutionary Genetic Analysis software MEGA v11 [86]. To confirm the identification of *B. pfeifferi* snails, phylogenetic trees were constructed using both Maximum Likelihood (ML) and Neighbor-Joining (NJ) methods in MEGA v.11. The ML tree was inferred under the General Time Reversible model with a gamma distribution (GTR+Γ), which was selected as the best-fit model. The NJ tree was generated using the Maximum Composite Likelihood (MCL) method. For both approaches, node support was assessed with 1000 bootstrap replicates to evaluate the reliability of inferred phylogenetic relationships, with ML and NJ bootstrap values given in the following order: ML/NJ. This was performed using a combined *COI* and *16S* data, and our samples were compared to different *Biomphalaria* spp. GenBank references (Supplementary Table S3).

#### 4.4.2. Haplotype and Population Genetic Analysis

The *COI* and *16S* rRNA datasets were analyzed separately, with all sequence alignments performed excluding gaps. Haplotype identification for each gene was conducted using DnaSP v5.10.01 [87], and genetic diversity parameters among *B. pfeifferi* populations were assessed using pairwise distances and diversity indices. These included the number of segregating sites (S), number of haplotypes (H), haplotype diversity (Hd), and nucleotide diversity (π). The average number of pairwise nucleotide differences within populations (K) was estimated using the Jukes–Cantor correction [88]. To assess signals of demographic history and population expansion, neutrality tests were performed separately for each population and the overall dataset. These included Tajima’s D [89], Fu and Li’s D and F tests [90], and Fu’s Fs neutrality test [91]. Genetic differentiation between populations was evaluated by calculating pairwise fixation indices (Fst), interpreted according to Wright’s criteria: low (Fst < 0.05), moderate (0.05–0.15), high (0.15–0.25), and very high (Fst > 0.25) [44,92]. Gene flow, expressed as the number of migrants per generation (Nm), was also estimated and categorized as low (Nm < 1), high (1–4), or very high (Nm > 4) [93]. Next, a TCS haplotype network was generated using PopArt v1.7 [94] to visualize reticulate relationships among *COI* and *16S* haplotypes from Gezira State and to compare them with other African *B. pfeifferi* sequences obtained from GenBank (Supplementary Tables S2–S5). The *B. pfeifferi* sequences generated in this study were deposited in GenBank under the following accession numbers: *COI* (SAMN39626448-SAMN39626452) and *16S* (SAMN39626453-SAMN39626457/SAMN40348294-SAMN40348298; Supplementary Tables S4 and S5).

### 4.5. PCR Infection Detection

All *B. pfeifferi* DNA extracts were tested for *S. mansoni* infection utilizing the Sm^F/R^ primer set designed by Sandoval et al. [33] (Table 6). All PCR reactions were performed using 11 μL of TianGEN 2× Taq PCR Mix buffer (0.1 U Taq polymerase/µL, 500 µM dNTPs, 20 mM Tris-HCl, 100 mM KCl, 3 mM MgCl_2_) with 5 μL of ddH2O, 1 μL of forward primer, 1 μL of reverse primer, and 2 μL of DNA template, giving a total volume of 20 µL. One negative (water) and positive control (*S. mansoni* DNA) were included. The PCR reaction mixture and cycling conditions followed precisely as described previously [33]. However, the Sm^F/R^ annealing temperature used in this study was 61 °C, and the DNA template used was 2 μL. In order to confirm the presence of a 350 bp band size for *S. mansoni*, PCR products were run on a 2% agarose gel containing GelStain C056 (1000× code #GS1014, Lot #P40910) and visualized under UV light. PCR products were purified and sequenced (Illumina Miseq) by Sangon Biotechnology Company Ltd. (Shanghai, China). After editing, Sm^F/R^ sequences were approximately 314–320 bp in length and were identified using the NCBI BLAST (the National Center for Biotechnology Information) tool (https://blast.ncbi.nlm.nih.gov) to find the most similar sequences on the GenBank Nucleotide Database. All Sm^F/R^ (*28S* rDNA) sequences generated in this study were added to GenBank under Bio-project (accession numbers: SAMN38735380-SAMN38735385).

## 5. Conclusions

This study provides the first combined assessment of genetic diversity and *Schistosoma mansoni* infection in *Biomphalaria pfeifferi* snails from Gezira State, Sudan. Mitochondrial markers indicated repeated bottlenecks and expansions driven by seasonal environmental fluctuations, with the *COI* showing strong population subdivision, and the *16S* reflecting within-population homogeneity due to selfing and inbreeding. Molecular PCR detection proved far more sensitive than cercarial shedding, revealing a higher prevalence of infection. Infected snails were confined to Hasahisa and occurred across multiple haplotypes, suggesting no genetic barrier to parasite establishment. Our results suggest that higher haplotype diversity may facilitate greater compatibility with *S. mansoni* and enhance local transmission potential. The restriction of infections to a single site highlights the focal nature of transmission and the role of localized ecological drivers. Incorporating molecular diagnostics into routine surveys would prevent underestimation of prevalence and improve xenomonitoring accuracy. Future studies using multi-marker and genomic approaches, coupled with expanded sampling across Sudan, are needed to clarify host–parasite interactions and support more targeted schistosomiasis control strategies.

## Figures and Tables

**Table 1:** Haplotype diversity and neutrality parameters of Sudanese *Biomphalaria pfeifferi* snails using the *COI* (527 bp) and *16S* (387 bp) gene fragments.

Gene	Site	H	S	K	Hd ±SD	HV	π (JC)± SD	Fu’ Fs	Fu Li’ D	Fu Li’ F	Tajima’s D	Theta Θ
*COI*	Overall	5	41	5.387	0.5 ± 0.022	0.0005	0.01 ± 0.0007	13.62	−5.01 **	−3.91 **	−0.867	0.014
	EG	3	31	2.80	0.191 ± 0.09	0.01	0.0055 ± 0.003	5.57	−2.84 *	−3.17 *	−2.36 **	0.015
	GW	1	0	0	-	-	-	-	-	-	-	-
	HA	2	3	0.2	0.067 ± 0.06	0.004	0.0003 ± 0.0003	0.135	−2.68 *	−2.79 *	−1.731	0.001
	NU1	1	0	0	-	-	-	-	-	-	-	-
	NU3	1	0	0	-	-	-	-	-	-	-	-
	SG	1	0	0	-	-	-	-	-	-	-	-
	MA	1	0	0	-	-	-	-	-	-	-	-
*16S*	Overall	10	84	2.056	0.11± 0.035	0.0012	0.008 ± 0.003	−0.043	−2.57 ***	−3.16 **	−2.710 ***	0.051
	EG	1	0	0	-	-	-	-	-	-	-	-
	GW	1	0	0	-	-	-	-	-	-	-	-
	HA	6	72	9.40	0.31 ± 0.109	0.012	0.035 ± 0.013	8.825	0.031	−0.69	−1.84 *	0.052
	NU1	4	18	1.32	0.25 ± 0.102	0.01	0.004 ± 0.003	1.38	−4.24 **	−4.3 ***	−2.425 ***	0.014
	NU3	2	1	0.06	0.067 ± 0.061	0.004	0.0002 ± 0.0002	−1.21	−1.68	−1.76	−1.147	0.001
	SG	1	0	0	-	-	-	-	-	-	-	-
	MA	1	0	0	-	-	-	-	-	-	-	-

Note: Statistical significance: * *p* < 0.05; ** *p* < 0.02; *** *p* < 0.01. EG: East Gezira; GW: Greater Wadmedani; HA: Hasahisa; NU: North Umelgura; SG: South Gezira; MA: Managil; H: number of haplotypes; S: number of segregating (polymorphic/variable) sites; K: average number of pairwise nucleotide differences; Hd: haplotype diversity; HV: haplotype diversity variance; SD: standard deviation; π: nucleotide diversity; JC: Jukes–Cantor correction; *Ɵ* theta per site.

**Table 2:** Estimation of pairwise population fixation index (Fst) comparisons of genetic differentiation among *Biomphalaria pfeifferi* populations using the *COI* and *16S* gene fragments.

(A) *COI* Gene Fragment							
	EG	GW	HA	NU1	NU3	SG	MA
EG	-						
GW	0.86	-					
HA	0.85	0	-				
NU1	0.02	1	0.99	-			
NU3	0.02	1	0.99	0	-		
SG	0.02	1	0.99	0	0	-	
MA	0.9	1	0.99	1	1	1	-
Overall Fst = 0.94, Overall Nm = 0.03							
**(B) *16S* Gene Fragment**							
	**EG**	**GW**	**HA**	**NU1**	**NU3**	**SG**	**MA**
EG	-						
GW	0	-					
HA	0.05	0.05	-				
NU1	0.01	0.01	0.04	-			
NU3	0	0	0.05	0.01	-		
SG	0	0	0.05	0.01	0	-	
MA	0	0	0.05	0.01	0	0	-
Overall Fst = 0.04, Overall Nm = 5.72							

Note: EG: East Gezira; GW: Greater Wadmedani; HA: Hasahisa; NU1: North Umelgura site 10; NU3: North Umelgura site 12; SG: South Gezira; MA: Managil; Nm: gene flow.

**Table 3:** Analysis of Molecular Variance (AMOVA) for *Biomphalaria pfeifferi COI* and *16S* haplotypes.

	Sum of Variation	Degree of Freedom (df)	Sigma Squared (σ^2^)	%Variation	Fixation Index (ΦST) *
*COI*					0.9 (*p* > 0.001)
Among populations	4203.261	6	25.616	84.6	
Within populations	724.800	156	4.646	15.4	
Total	4928.061	162	30.262		
*16S*					0.04 (*p* > 0.001)
Among populations	556.691	6	1.727	3.6	
Within populations	7154.567	156	45.863	96.4	
Total	7711.258	162	47.590		

* (ΦST): Significance (1000 permutations).

**Table 4:** Infection prevalence of *Biomphalaria pfeifferi* snails determined by the cercarial shedding method and PCR detection using Sm^F/R^ primers.

Site (ID)	Number of *B. pfeifferi* Tested			
	Collected	Cercaria Shedding	PCR (Sm^F/R^)	Type of Cercariae
SG (S3)	10	0	0	None
GW (S6)	38	0	0	None
NU1 (S10)	31	0	0	None
NU3 (S12)	30	16.6% (5/30)	0	*Echinostome* (n:5) and *Cotylomicrocercous* spp. (n:4)
EG (S13)	66	1.5% (1/66)	0	*Apharyngostrigea* sp.
HA (S19)	39	5.1% (2/39)	40% (12/30)	*S. mansoni*
MA (S23)	3	0	0	None
Overall infection rate		3.6% (8/219)	7.4% (12/163) *	

Note: * No. of extracted snails was 30 samples per area for East Gezira, North Umelgura, and Hasahisa; 10 for South Gezira; and 3 for Managil localities (total 163). *S. mansoni*: *Schistosoma mansoni.*

**Table 5:** Summary of non-perennial canal collection sites in Gezira State, Sudan, where Biomphalaria pfeifferi snails were found, including the number of DNA-extracted specimens and GPS coordinates.

Locality/Administrative Unit	Site ID		No. DNA Extracted (n = 163)	Latitude	Longitude
South Gezira/Barakat	S3: Barakat	(SG)	10	14.357	33.526
Greater Wadmedani	S6: Atraa	(GW)	30	14.445	33.487
North Umelgura/Elhediba	S10: Elhediba	(NU1)	30	14.484	33.658
	S12: Elhediba	(NU3)	30	14.484	33.657
East Gezira/Elgineid	S13: Elgineid	(EG)	30	14.866	33.277
Hasahisa/Wadelfadni	S19: Wadelfadni	(HA)	30	14.670	33.343
Managil/Eboud	S23: Eboud/Alnegeer village	(MA)	3	14.230	33.173

**Table 6:** Primer set sequences used for the amplification of *COI* and *16S rRNA* mitochondrial DNA for *Biomphalaria pfeifferi* molecular identification and the Sm^F/R^ primer set used for *Schistosoma mansoni 28S rRNA* amplification.

Primer Name	Sequence (5′→3′)	Primer Length (bp)	Annealing Temperature (°C)
LCO 1490	GGT CAA CAA ATC ATA AAG ATA TTG G	25	45
HCO 2198	TAA ACT TCA GGG TGA CCA AAA AAT CA	26	
16Sar	CTT CTC GAC TGT TTA TCA AAA ACA	24	48
16Sbr	GCC GGT CTG AAC TCA GAT CAT	21	
SmF	GAG ATC AAG TGT GAC AGT TTT GC	23	61
SmR	ACA GTG CGC GCG TCG TAA GC	20	

## Data Availability

All data is given within the manuscript itself. Sequence data is provided in GenBank accession numbers SAMN39626448-SAMN39626452 (*COI*), SAMN39626453-SAMN39626457/SAMN40348294-SAMN40348298 (*16S*), and SAMN38735380-SAMN38735385 (*28S* Sm^F/R^).

## Supplementary Materials

The following supporting information can be downloaded at: https://www.mdpi.com/article/10.3390/ijms26199567/s1. References [2,15,21,23,25,26,28,45,95,96] are cited in Supplementary Materials.

## Abbreviations

*COI*: Cytochrome oxidase subunit I; *16S* rRNA: *16S* ribosomal RNA subunit; *28S* rDNA: *28S* ribosomal DNA subunit; *ITS*: Internal transcribed spacer; Fst: Pairwise fixation index; Nm: Gene flow/number of migrants; GPS: Global Positioning System; GIS: Geographic Information System; PCR: Polymerase chain reaction; mL: Milliliter; mM: Millimolar; mg: Milligram; μL: Microliter; bp: Base pair.
