# Supplementary material for: Genetic Diversity and Infection Prevalence of Biomphalaria pfeifferi (Krauss, 1848), the Intermediate Snail Host of Schistosoma mansoni in Gezira State, Sudan
Source: Int J Mol Sci. 2025 Sep 30;26(19):9567. doi: 10.3390/ijms26199567 (PMC12524667; doi:10.3390/ijms26199567)
Supplement: Supplementary file 1 [file ijms-26-09567-s001.zip › Supplement Figures V2.pdf]

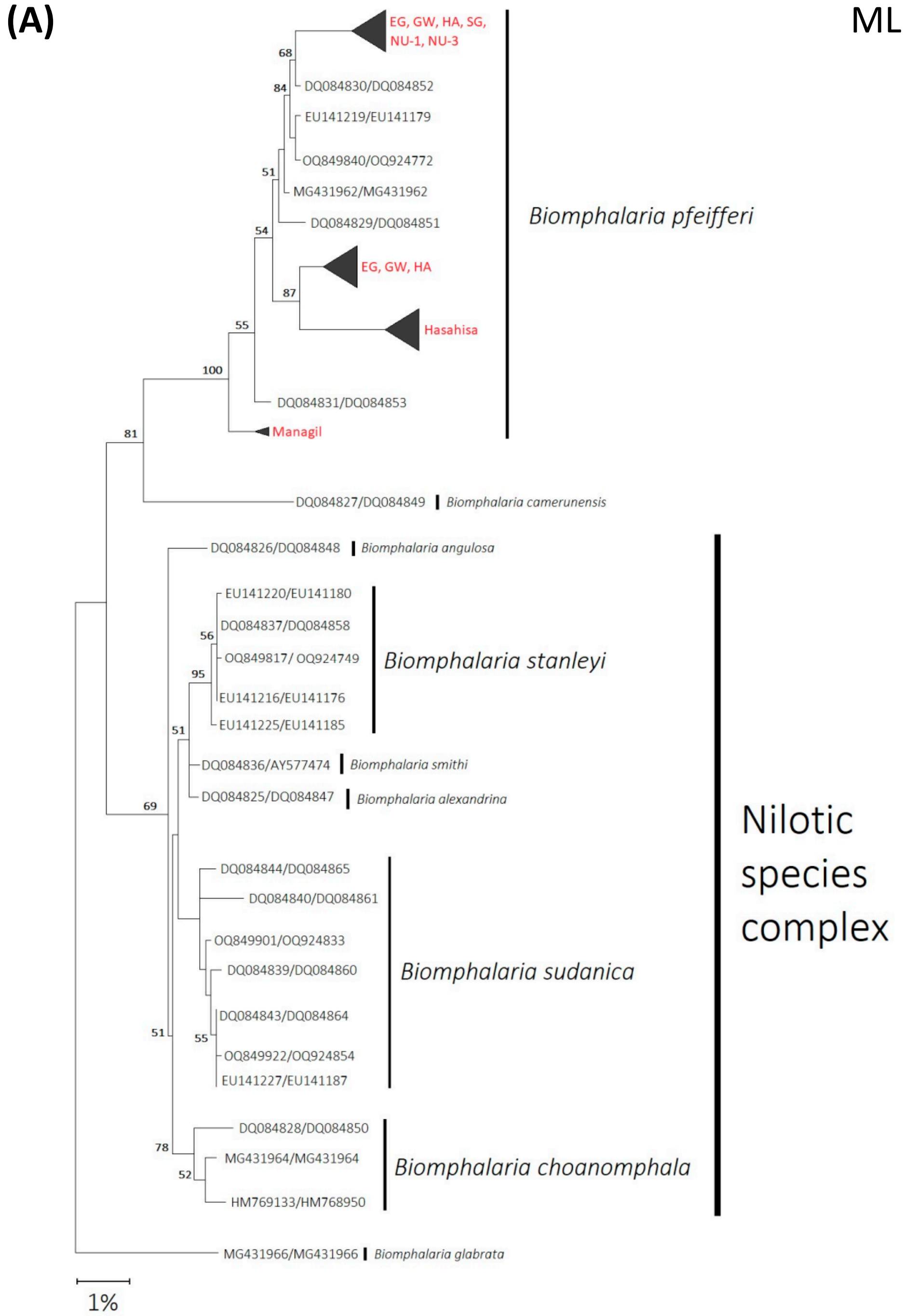

(A)
ML
EG, GW, HA, SG, NU-1, NU-3
DQ084830/DQ084852
EU141219/EU141179
OQ849840/OQ924772
MG431962/MG431962
DQ084829/DQ084851
EG, GW, HA
Hasahisa
DQ084831/DQ084853
Managil
Biomphalaria pfeifferi
DQ084827/DQ084849
Biomphalaria camerunensis
DQ084826/DQ084848
Biomphalaria angulosa
EU141220/EU141180
DQ084837/DQ084858
OQ849817/ OQ924749
EU141216/EU141176
EU141225/EU141185
Biomphalaria stanleyi
DQ084836/AY577474
Biomphalaria smithi
DQ084825/DQ084847
Biomphalaria alexandrina
DQ084844/DQ084865
DQ084840/DQ084861
OQ849901/OQ924833
DQ084839/DQ084860
DQ084843/DQ084864
OQ849922/OQ924854
EU141227/EU141187
Biomphalaria sudanica
DQ084828/DQ084850
MG431964/MG431964
HM769133/HM768950
Biomphalaria choanomphala
Nilotic species complex
MG431966/MG431966
Biomphalaria glabrata
1%

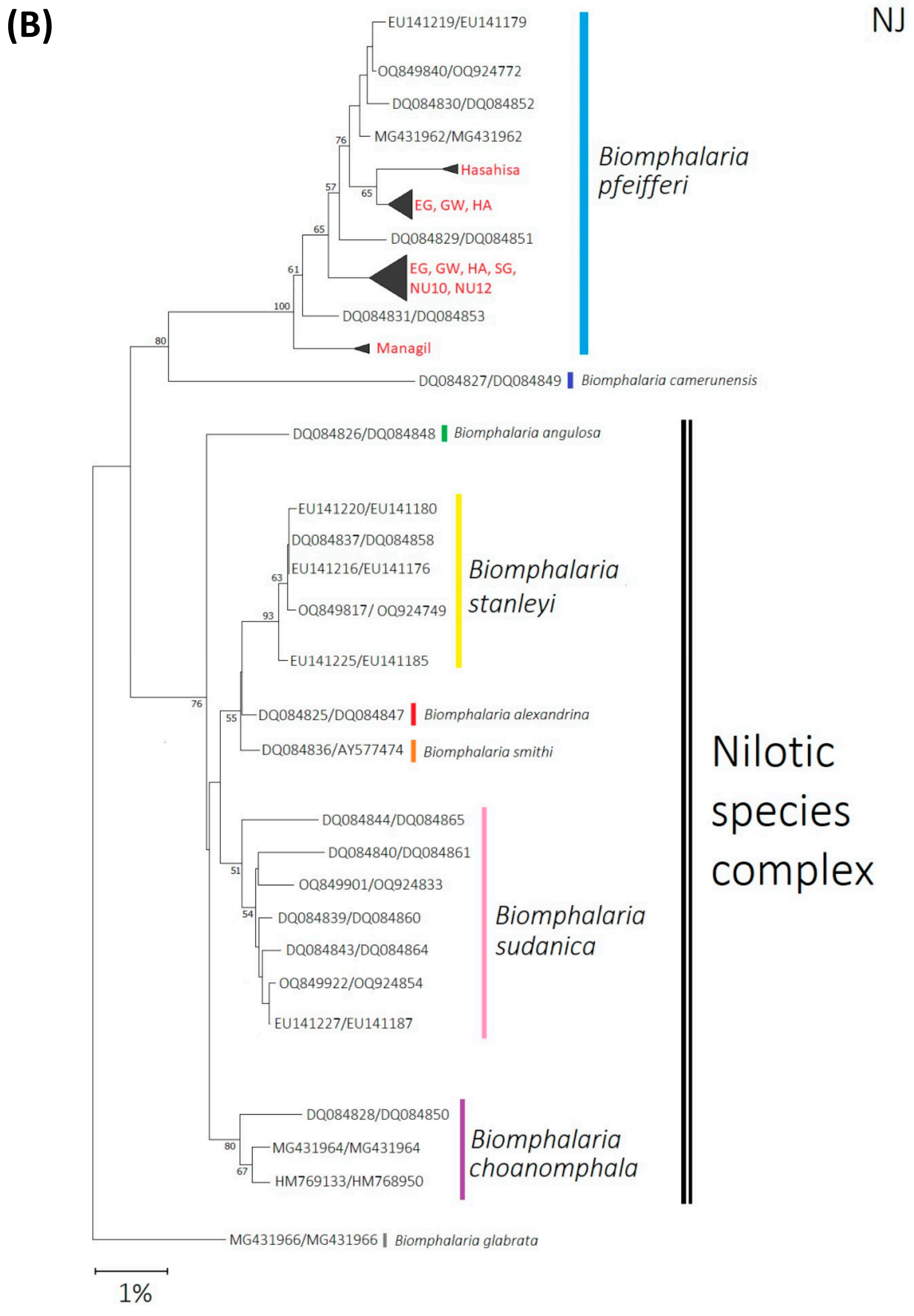

Supplementary Figure S1 The individual (A) Maximum Likelihood (ML) and (B) Neighbor-Joining (NJ) trees of African *Biomphalaria* species based on concatenated *COI* (465 bp) and *16S rRNA* (322 bp) gene fragments. Phylogenetic reconstruction was performed using the GTR+Γ model for ML and the Maximum Composite Likelihood (MCL) method for NJ, with trees rooted on *B. glabrata*. Red labels indicate *B. pfeifferi* sequences generated in this study. Numbers on branches show bootstrap support values (1000 replicates); values <50% are not displayed. The scale bar represents 1% sequence divergence.

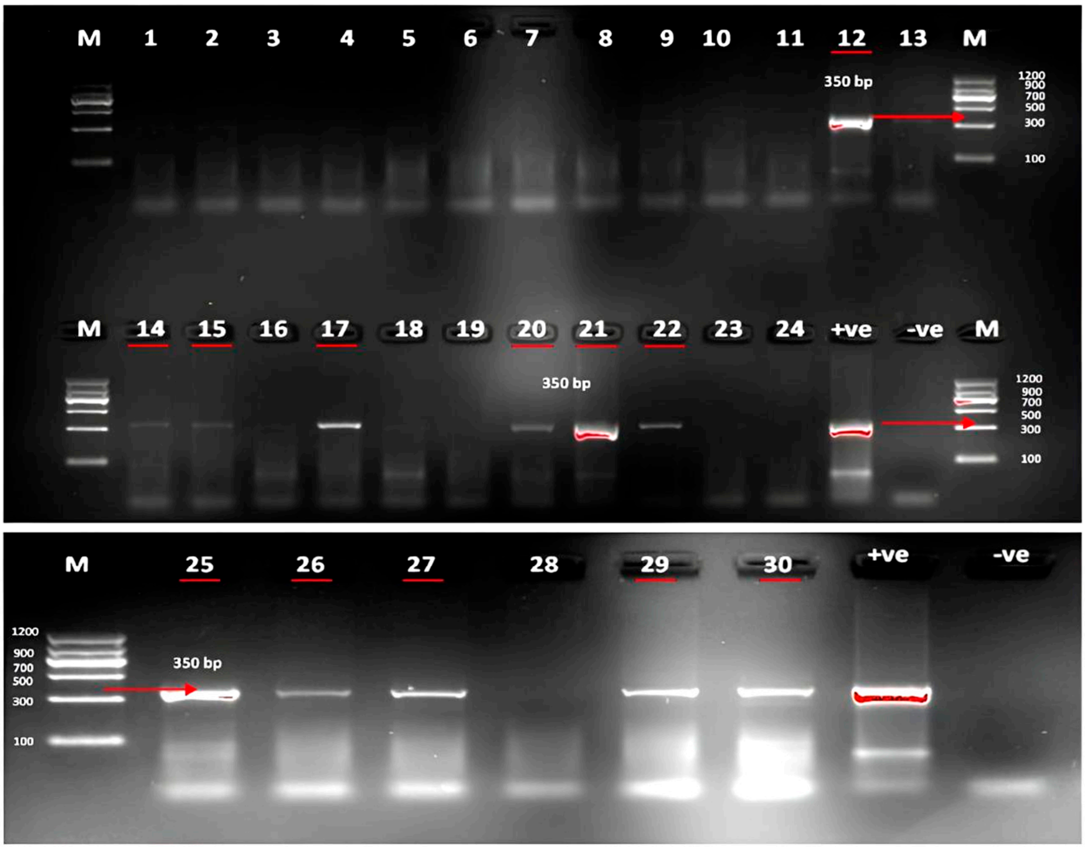

Supplementary Figure S2 PCR amplification of *S. mansoni* infected *B. pfeifferi* snails using the $Sm^{F/R}$ primer set. All samples are from Hasahisa (lanes 1–30), with lanes 12, 14, 15, 17, 20-22, 25-27, 29 and 30 showing a ~350 bp diagnostic band (underlined in red). PCR products were visualized on a 2% agarose gel.

Note: Lane M = DNA ladder (100 - 1200 bp); Lane +ve = positive control (*S. mansoni* DNA); and Lane –ve = negative control (water).
