# Supplementary material for: Genetic Diversity and Infection Prevalence of Biomphalaria pfeifferi (Krauss, 1848), the Intermediate Snail Host of Schistosoma mansoni in Gezira State, Sudan
Source: Int J Mol Sci. 2025 Sep 30;26(19):9567. doi: 10.3390/ijms26199567 (PMC12524667; doi:10.3390/ijms26199567)
Supplement: Supplementary file 1 [file ijms-26-09567-s001.zip › Supplement Tables V2.pdf]

Supplementary Table S1. Haplotype composition of *Biomphalaria pfeifferi* populations from the seven sampling sites in Gezira State, Sudan.

COI	H1	H2	H3	H4	H5
S3 (SG)	10	0	0	0	0
S6 (GW)	0	30	0	0	0
S10 (NU1)	30	0	0	0	0
S12 (NU3)	30	0	0	0	0
S13 (EG)	27	2	1	0	0
S19 (HA)	0	29	0	1	0
S23 (MA)	0	0	0	0	3

16S	H1	H2	H3	H4	H5	H6	H7	H8	H9	H10
S3 (SG)	10	0	0	0	0	0	0	0	0	0
S6 (GW)	30	0	0	0	0	0	0	0	0	0
S10 (NU1)	26	0	0	0	0	0	1	2	1	0
S12 (NU3)	29	0	0	0	0	0	0	0	0	1
S13 (EG)	30	0	0	0	0	0	0	0	0	0
S19 (HA)	25	1	1	1	1	1	0	0	0	0
S23 (MA)	3	0	0	0	0	0	0	0	0	0

Supplementary Table S2. Estimates of pairwise evolutionary divergence between sequences in **(A)** *COI* haplotypes and **(B)** *16S* haplotypes using the Maximum Composite Likelihood model.

(A) ***COI***

	H1	H2	H3	H4	H5
**H1**	-				
**H2**	0.019	-			
**H3**	0.053	0.068	-		
**H4**	0.025	0.006	0.074	-	
**H5**	0.027	0.023	0.075	0.029	-

(B) ***16S***

	H1	H2	H3	H4	H5	H6	H7	H8	H9	H10
**H1**	-									
**H2**	0.145	-								
**H3**	0.192	0.064	-							
**H4**	0.064	0.116	0.145	-						
**H5**	0.006	0.145	0.184	0.064	-					
**H6**	0.003	0.141	0.188	0.067	0.009	-				
**H7**	0.054	0.187	0.22	0.116	0.060	0.057	-			
**H8**	0.003	0.149	0.196	0.067	0.009	0.006	0.050	-		
**H9**	0.012	0.160	0.203	0.077	0.018	0.015	0.057	0.009	-	
**H10**	0.003	0.149	0.195	0.067	0.009	0.006	0.057	0.006	0.015	-

Supplementary Table S3. GenBank accession numbers and corresponding references for the *COI* and *16S* phylogenetic tree.

Accession no.		Species	Reference
COI	***16S rRNA***		
DQ084836	AY577474	*smithi*	Jørgensen et al. (1)
DQ084825	DQ084847	*alexandrina*	
DQ084826	DQ084848	*angulosa*	
DQ084827	DQ084849	*camerunensis*	
DQ084828	DQ084850	*choanomphala*	
DQ084829	DQ084851	*pfeifferi*	
DQ084830	DQ084852	*pfeifferi*	
DQ084831	DQ084853	*pfeifferi*	
DQ084837	DQ084858	*stanleyi*	
DQ084839	DQ084860	*sudanica*	
DQ084840	DQ084861	*sudanica*	
DQ084843	DQ084864	*sudanica*	
DQ084844	DQ084865	*sudanica*	
EU141216	EU141176	*stanleyi*	Plam et al. (2)
EU141219	EU141179	*pfeifferi*	
EU141220	EU141180	*stanleyi*	
EU141225	EU141185	*stanleyi*	
EU141227	EU141187	*sudanica*	
EU141228	EU141188	*sudanica*	
MG431962	MG431962	*pfeifferi*	Zhang et al. (3)
MG431964	MG431964	*choanomphala*	
MG431966	MG431966	*glabrata*	
HM769133	HM768950	*choanomphala*	Standley et al. (4)
OQ849817	OQ924749	*stanleyi*	Andrus et al. (5)
OQ849828	OQ924850	*choanomphala*	
OQ849840	OQ924772	*pfeifferi*	
OQ849901	OQ924833	*sudanica*	
MG431966	MG431966	*glabrata*	Zhang et al. (3)

Supplementary Table S4. GenBank accession numbers and corresponding references for *B. pfeifferi* COI gene analyzed in this study.

Accession no. for COI	Country	Reference
AF199099	Senegal	Martin and Rollinson [unpublished]
AF199100	Mali	
DQ084833	Uganda	Jorgensen et al. (1)
MG780150	Sudan	Abe [unpublished]
MG780152	Sudan	
MG780182	Zimbabwe	Mutsaka-Makuvaza et al. (9)
OL423116	Kenya	Laidemitt et.al. (6)
OQ216742 (H1)	Malawi	Alharbi et al. (7)
OQ216743 (H2)		
OQ216744 (H3)		
OQ216745 (H4)		
OQ216746 (H5)		
OR880348	Malawi	Archer and Stothard, [8]
SAMN39626448 (H1)	Sudan	This study
SAMN39626449 (H2)		
SAMN39626450 (H3)		
SAMN39626451 (H4)		
SAMN39626452 (H5)		

Supplementary Table S5. GenBank accession numbers and corresponding references for *B. pfeifferi 16S* gene analyzed in this study.

Accession no. for 16S	Country	Reference
AY030193	Senegal	Dejong et al. (10)
AY030194	Cameroon	
AY030195	Sudan	
AY030196	Madagascar	
AY126600	Zimbabwe	
AY198052	Kenya	
AY198060	Zambia	
AY198065	Quate devoir	
AY198075	Sudan	
DQ084852	Kenya	Jorgensen et al. (1)
DQ084857	Sudan	
EU141179	Uganda	Plam et al. (2)
OQ924803	Uganda	Andrus et al. (5)
SAMN39626453 (H1)	Sudan	This study
SAMN39626454 (H2)		
SAMN39626455 (H3)		
SAMN39626456 (H4)		
SAMN39626457 (H5)		
SAMN40348294 (H6)		
SAMN40348295 (H7)		
SAMN40348296 (H8)		
SAMN40348297 (H9)		
SAMN40348298 (H10)		

References

1. Jorgensen A, Kristensen TK, Stothard JR. Phylogeny and biogeography of African *Biomphalaria* (Gastropoda: Planorbidae), with emphasis on endemic species of the great East African lakes. Zoological Journal of the Linnean Society. 2007;151(2):337–49.
2. Plam M, Jørgensen A, Kristensen T, Madsen H. Sympatric *Biomphalaria* species (Gastropoda: Planorbidae) in Lake Albert, Uganda, show homoplasies in shell morphology. African Zoology. 2008 01;43:34–44.
3. Zhang SM, Bu L, Laidemitt MR, Lu L, Mutuku MW, Mkoji GM, et al. Complete mitochondrial and rDNA complex sequences of important vector species of *Biomphalaria*, obligatory hosts of the human-infecting blood fluke, *Schistosoma mansoni.* Scientific Reports. 2018 May 9;8(1):7341.
4. Standley CJ, Goodacre SL, Wade CM, Stothard JR. The population genetic structure of *Biomphalaria choanomphala* in Lake Victoria, East Africa: implications for schistosomiasis transmission. Parasites & Vectors. 2014;7:1–10.
5. Andrus PS, Stothard JR, Wade CM. Seasonal patterns of *Schistosoma mansoni* infection within Biomphalaria snails at the Ugandan shorelines of Lake Albert and Lake Victoria. PLOS Neglected Tropical Diseases. 2023;17(8):e0011506.
6. Laidemitt M, Gleichsner A, Ingram C, Gay S, Reinhart E, Mutuku M, et al. Host preference of field-derived *Schistosoma mansoni* is influenced by snail host compatibility and infection status. Ecosphere. 2022 08;13.
7. Alharbi MH, Condemine C, Hesketh J, Kayuni SA, Arme TM, Archer J, et al. Biomphalaria pfeifferi (Gastropoda: Planorbidae) in Lake Malawi and Upper Shire River, Mangochi District, Malawi: distribution, genetic diversity and pre-patent schistosome infections. Tropical Medicine and Infectious Disease. 2023; 8(2):126.
8. Archer J, Cunningham LJ, Juhász A, Jones S, O'Ferrall AM, Rollason S, et al. Molecular epidemiology and population genetics of *Schistosoma mansoni* infecting school-aged children situated along the southern shoreline of Lake Malawi, Malawi. PLOS Neglected Tropical Diseases. 2024;18(10):e0012504.
9. Mutsaka-Makuvaza MJ, Zhou XN, Tshuma C, Abe E, Manasa J, Manyangadze T, et al. Genetic diversity of *Biomphalaria pfeifferi*, the intermediate host of *Schistosoma mansoni* in Shamva district, Zimbabwe: Role on intestinal schistosomiasis transmission. Molecular Biology Reports. 2020;47:4975–87.
10. Dejong RJ, Morgan JA, Wilson WD, Al-Jaser MH, Appleton CC, Coulibaly G, et al. Phylogeography of *Biomphalaria glabrata* and *B. pfeifferi*, important intermediate hosts of *Schistosoma mansoni* in the New and Old World tropics. Mol Ecol. 2003 Nov;12(11):3041–56.
